# Evaluating the Contribution of North American Zoos and Aquariums to Endangered Species Recovery

**DOI:** 10.1038/s41598-018-27806-2

**Published:** 2018-06-28

**Authors:** Judy P. Che-Castaldo, Shelly A. Grow, Lisa J. Faust

**Affiliations:** 1 Alexander Center for Applied Population Biology, Lincoln Park Zoo, Chicago, IL USA; 2 Association of Zoos and Aquariums, Silver Spring, MD USA

## Abstract

The challenge of recovering threatened species necessitates collaboration among diverse conservation partners. Zoos and aquariums have long partnered with other conservation organizations and government agencies to help recover species through a range of *in situ* and *ex situ* conservation projects. These efforts tend to be conducted by individual facilities and for individual species, and thus the scope and magnitude of these actions at the national level are not well understood. Here we evaluate the means and extent to which North American zoos and aquariums contribute to the recovery of species listed under the U.S. Endangered Species Act (ESA), by synthesizing data from federal recovery plans for listed species and from annual surveys conducted by the Association of Zoos and Aquariums. We found that in addition to managing *ex situ* assurance populations, zoos frequently conduct conservation research and field-based population monitoring and assessments. Cooperatively managed populations in zoos tend to focus on species that are not listed on the ESA or on foreign listings, and thus it may be beneficial for zoos to manage more native threatened species. Our results highlight the existing contributions, but also identify additional opportunities for the zoo community to help recover threatened species.

## Introduction

Due to the magnitude and complexity of the global extinction crisis, successful species conservation will require the engagement of all potential partners: state and federal agencies, non-governmental organizations, local communities and resource users, industry stakeholders, and wildlife managers^1^. These diverse partners each bring unique perspectives, expertise, and resources, not all of which will be appropriate or necessary in every case. However, a clear understanding of the potential contributions of each partner will help to identify the most relevant entities to call upon in each case.

Zoos and aquariums (hereafter, “zoos”) are becoming more broadly recognized as important partners for conserving threatened species^2,3^. There is a long history of zoos engaging in species recovery, from the American bison and California condor to the black-footed ferret and Panamanian golden frog^4^. However, the role of zoos in species conservation has often focused on *ex situ* species management, in particular *ex situ* breeding^5,6^. For example, the Conservation Measures Partnership’s Actions Classification^7^ identifies 30 distinct types of conservation actions, but specifies a role for zoos in only two of those (*ex situ* conservation, outreach and communications). The conservation value of *ex situ* breeding has also been somewhat controversial, with views ranging from it being a last resort that diverts resources from *in situ* efforts^8^, to part of a continuum of management actions for threatened species^9^. Even when *ex situ* breeding is acknowledged as part of the conservation strategy, the ability of zoos to sustain demographically and genetically viable populations for the long-term has been questioned^10,11^. Undoubtedly these issues and concerns must continue to be explored, but zoos also contribute to other conservation efforts beyond *ex situ* breeding^12–14^.

Several publications have explored generally how zoos contribute to species conservation, discussing both *in situ* and *ex situ* actions. *Ex situ* actions can directly target the species (*e*.*g*., *ex situ* population management, rehabilitation, gene banking)^7^, or indirectly support conservation through public outreach, biological and veterinary research, and fundraising for other organizations and projects^3,14,15^. *In situ* actions can include engaging and educating communities in the species’ native range, protecting and restoring habitat, supplying animals and/or staff for reintroductions, and field-based monitoring^3,15^. Although there are many case studies of these individual actions, the extent to which zoos contribute to conservation through these actions is not well understood. One study has evaluated the impacts of a subset of *in situ* conservation projects branded by the World Association of Zoos and Aquariums^16^, and another summarized the number of breeding and reintroduction projects for threatened species conducted by four Canadian zoos^12^. Thus far, no study has quantified both the *in situ* and *ex situ* conservation actions conducted by zoos at a national scale.

In the U.S., all institutions accredited by the Association of Zoos and Aquariums (AZA) include species conservation as a key part of their missions, in accordance with accreditation standards. To fulfill this part of their missions, zoos carry out an array of *in situ* and *ex situ* initiatives^4^, and collaborate with other conservation organizations and government agencies. This includes the agencies [U.S. Fish and Wildlife Service (USFWS) and National Oceanic and Atmospheric Administration (NOAA) Fisheries] that implement the U.S. Endangered Species Act (ESA), which was enacted in 1973 to protect threatened species through both extinction prevention and recovery actions^17^. However, the extent and scope of these zoo conservation efforts have not been systematically evaluated beyond annual reports within the zoo community.

The goal of this study was to evaluate the contribution of zoos to the recovery of threatened species in the U.S. by quantifying and summarizing their conservation activities. Our analysis consisted of three parts: (1) Summarize the management actions for which zoos are the responsible parties, based on data from federal recovery plans for listed species; (2) Summarize the recent conservation activities reported by AZA-accredited facilities in responses to the association’s annual field conservation and research surveys; and (3) Quantify the number of listed species that currently have managed populations in AZA facilities in order to identify additional opportunities for species conservation. Using multiple datasets allowed us to compare the contributions as self-reported by AZA facilities against those as recognized by the agencies responsible for implementing the ESA. Due to the scope of our study, we did not aim to quantify the impacts of these conservation activities, although it would be a valuable assessment that could be implemented following the methods of Mace *et al*.^18^.

In this study we focused on the terrestrial (including invertebrate and amphibian) and avian species listed under the ESA as of February 2017. Therefore, the large number of zoo conservation projects on marine and aquatic species, and the small number on plant species, were outside the scope of this assessment. Zoo conservation projects involving species with other risk statuses (*e*.*g*., Candidate, Under Review, or Proposed status under the ESA; state-listed; those ranked as Threatened (VU, EN, CR) or Extinct in the Wild (EW) under the IUCN Red List but not listed under the ESA) were also not represented in this assessment. Additionally, we focused on listed species whose native range included the U.S. (*i*.*e*., U.S. or U.S./foreign listings under the ESA; “U.S. listings” hereafter) in the first two parts of our analysis, but explored the overlap between both U.S. and foreign listings with managed zoo programs in the last section.

## Results

### Roles of Zoos and Aquariums in Recovery Plans

The ESA requires every listed species to have a recovery plan, which documents the management actions and the criteria that determine when the species can be delisted. We gathered recovery plan data from the USFWS Recovery Plan Ad Hoc Report database (http://ecos.fws.gov/ecp0/ore-input/ad-hoc-recovery-actions-public-report-input), by querying all recovery actions that list a zoo, aquarium, or AZA (“zoos”) as the responsible party. As of September 2016, the recovery plans for 73 listed species (15.1% of the 482 listings that have recovery plans) named zoos as responsible for at least one recovery action. Of these, we focused on the 54 terrestrial and avian animals (6 amphibians, 31 birds, 7 invertebrates, and 10 mammals) for this analysis. Forty-two of these species are currently listed as Endangered and eight as Threatened, one is not listed due to extinction but was a species of concern at the time of recovery planning (*Moho bishopi*), and three have been delisted since the plan was written due to recovery (*Urocyon littoralis* subspecies *littoralis*, *santacruzae*, and *santarosae*).

In total, there were 38 recovery plans (some plans included more than one species) that described 468 recovery actions for which zoos were the responsible party. These actions involved 39 individual zoos or aquariums, or else listed AZA as the responsible party (see Table S1 for complete list of institutions). We determined 11 keywords to represent the major types of conservation activities attributed to zoos (Table 1), which were derived through an iterative process. We started with 52 keywords used by AZA to categorize zoo conservation and science projects (see next section), and condensed them into 9 categories (*e*.*g*., anti-poaching/patrolling, disaster/emergency response, human-wildlife conflict, and wildlife trade were grouped into “threat mitigation”). We assigned these broader keywords to each recovery action based on the action descriptions from the plans, and added two keywords (fundraising, management/planning) to describe recovery actions that did not fit into existing keywords. In some cases multiple keywords were assigned to an action, resulting in a total of 605 keywords assigned.

**Table 1:** Keywords used to assign each recovery action or conservation project into categories.

Keyword	Description or examples
Assurance population	*Ex situ* breeding; establishing or maintaining *ex situ* populations to guard against extinction or serve as potential source for reintroduction
Education/outreach	Developing materials and displays; increasing awareness and partnerships
Fundraising	Seeking federal, state, and private grants; supporting other organizations
Habitat creation/restoration/protection	Purchasing habitat; creating nest sites; implementing restoration programs
Husbandry/veterinary care	Conducting physical exams; evaluating reproductive status; treating diseases
Management/planning	Decision making; evaluation; coordinating or serving on recovery teams or similar groups
Monitoring/assessments	Field-based population monitoring
Population augmentation	Activities affecting the *in situ* population (*e*.*g*., reintroduction of bred, head-started, or rehabilitated animals; translocation; release)
Rescue/rehab/sanctuary	Providing staff or facilities for rehabilitation and/or rescue
Research	Scientific studies in any area including behavior, demography, endocrinology, genetics, physiology, social sciences; field-based and zoo-based studies
Threat mitigation	Preventing or responding to threats (*e*.*g*., strandings, disease, predation, natural disasters)

**Figure 1:**
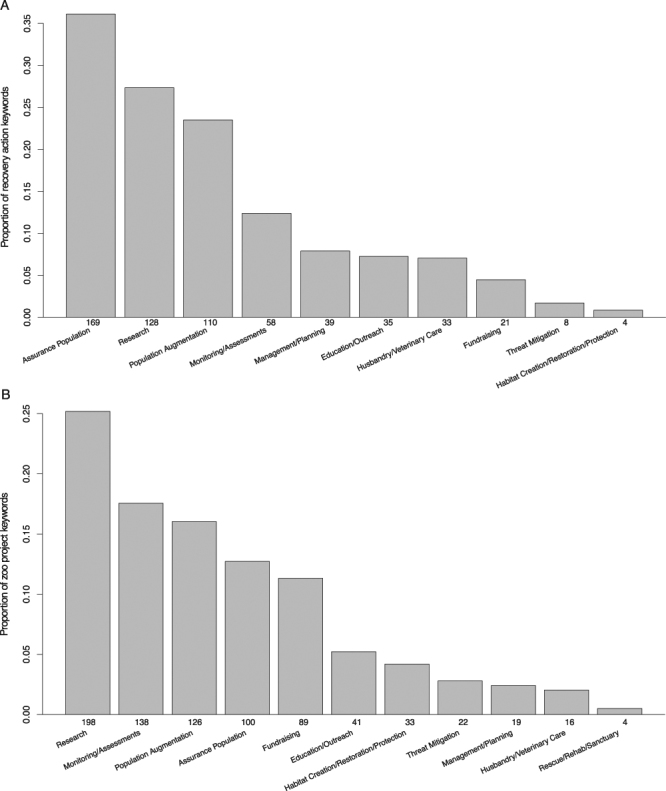
Conservation activities carried out by North American zoos and aquariums for species listed under the Endangered Species Act, sorted by type using 11 keywords. The number of instances of each keyword is shown at the base of the bars. (**A**) Distribution of the 468 recovery actions for which zoos and aquariums are the responsible party as described in recovery plans; a total of 606 keywords were assigned. (**B**) Distribution of the 644 field conservation and research project submissions by zoos to the 2013–2015 Annual Report on Conservation and Science (ARCS) survey; a total of 786 keywords were assigned.

The majority of recovery actions related to managing and/or maintaining an assurance population (36.1% of keywords), research (27.4%), and population augmentation (23.5%; Fig. 1A). Research included a broad range of topics relevant to species recovery, from investigating the impacts of contaminants, to modeling disease dynamics, to evaluating methods for habitat restoration. Besides population augmentation, other *in situ* recovery actions primarily consisted of population monitoring and assessments (12.4%), but there were also a small number of projects related to mitigating threats (1.7%) and to protecting and restoring habitat (0.9%). An unexpected type of zoo recovery action was management and planning (8.3%), which included projects that either involved or supported decision-making by the recovery team, such as coordinating program components, prioritizing tasks, or evaluating existing strategies. These tasks help to improve efficiency and flexibility and therefore can contribute greatly to the success of a conservation program. Other previously recognized contributions from zoos such as education and outreach^7,19^ and husbandry knowledge and veterinary care^13^ were also represented in recovery plans (7.5% and 7.1%, respectively). Finally, zoos contributed to conservation by providing project funds (4.5%), which were raised not only through visitor fees^8^ but also by securing state, federal, and private grants. The keyword related to providing rescue, rehabilitation, or sanctuary facilities did not apply to any zoo-based recovery actions described in these plans. However, they may be more likely to be included in plans for ESA-listed marine species (*e*.*g*., sea turtles).

**Figure 2:**
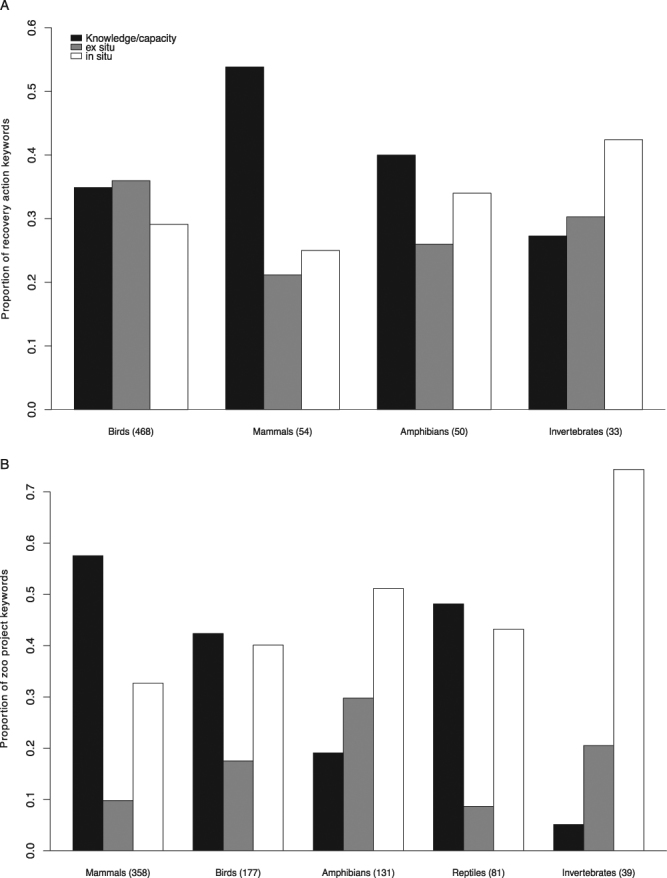
Conservation activities carried out by North American zoos and aquariums for species listed under the Endangered Species Act, by taxonomic group. Activities were aggregated into three categories based on the activity type keywords: conservation knowledge or capacity (research, education/outreach, management/planning, fundraising), *ex situ* (assurance population, husbandry/veterinary care, rescue/rehab/sanctuary), and *in situ* (population augmentation, monitoring/assessments, threat mitigation, and habitat creation/restoration/protection). The total instances of keywords for each taxonomic group are shown in parentheses. (**A**) Distribution of the 468 recovery actions for which zoos and aquariums are the responsible party from recovery plans; a total of 606 keywords were assigned. (**B**) Distribution of the 644 field conservation and research project submissions by zoos to the 2013–2015 Annual Report on Conservation and Science (ARCS) survey; a total of 786 keywords were assigned.

Recovery actions were distributed unevenly across taxa (Fig. 2A), with the majority of actions pertaining to birds (357 out of 468 actions). This was because the Revised Hawaiian Forest Birds Recovery Plan^20^ included a very similar set of up to 19 recovery actions for each of 19 different bird species (for a total of 289 recovery actions) that involved either the San Diego Zoological Society or the Honolulu Zoo. To compare recovery action types among taxonomic groups, we further clustered the 11 project keywords into three broader categories: *ex situ*, *in situ*, and knowledge/capacity. *Ex situ* included the projects related to animal care and management at zoos (i.e., assurance population, husbandry/veterinary care, rescue/rehabilitation/sanctuary), whereas *in situ* included projects that took place at the species’ native range (i.e., population augmentation, monitoring/assessments, threat mitigation, and habitat creation/restoration/protection). The remaining project types all focused on increasing biological knowledge or the capacity for conservation (i.e. research, education/outreach, management/planning, fundraising). For birds, all three categories of projects were similarly common, with a slightly lower proportion of *in situ* projects (Fig. 2A). In contrast, *in situ* projects were the most common category for invertebrates. Knowledge and capacity-building projects (primarily research) were the most common type of zoo recovery action for mammals and amphibians, accounting for 56% and 40% of their action keywords, respectively.

In addition to working with federal agencies in recovery programs, zoos also collaborate with other partners, including academic institutions, research institutions, or universities (collectively “academic institutions”) and other non-governmental organizations (NGOs). Thus we also examined the involvement of these two types of partners in the recovery actions that specified zoos as a responsible party. All four recovery actions related to habitat creation/restoration/protection listed either academic institutions (2 actions) or other NGOs (2 actions) as additional responsible parties, suggesting such field projects may require larger collaborations to implement. Academic institutions were involved in nearly half of the actions with research as a keyword (54 out of 128 actions), but did not collaborate with zoos as much on other types of recovery actions (<13% for all other types). Other NGOs partnered with zoos most frequently on actions related to assurance populations (26 out of 169 actions) and research (26 out of 128 actions), but proportionally they collaborated primarily on actions related to education and outreach (14 out of 35 actions) and threat mitigation (2 out of 8 actions).

Although recovery plans provide an official documentation of the extent to which zoos participate in recovery programs when the plans were created, they do not provide the full picture. Nearly one-third of all U.S. listed animals do not have a recovery plan (482 out of 710 listed animal species had plans as of September 2016), and finalized plans are rarely updated and therefore tend to exclude more recent or current projects. Additionally, a zoo’s involvement may not have been explicitly described as a recovery action, or only the primary holding facilities may have been identified when multiple institutions are involved.

### Conservation Activities Reported by Zoos and Aquariums

We next summarized zoo conservation activities based on the AZA’s field conservation and research surveys from 2013–2015. These surveys are used to produce the association’s Annual Report on Conservation and Science (ARCS; http://www.aza.org/annual-report-on-conservation-and-science). In the field conservation survey, AZA member institutions report only their conservation efforts that have direct impacts on animals and habitats in the wild. In the research survey, they report on any hypothesis-driven research conducted at these institutions or by their staff and the resulting publications. Response rates differed between surveys and years, with 86–92% of institutions responding for the field conservation survey and 52–64% responding for the research survey between 2013–2015. Although this dataset likely underrepresents the conservation and research projects in zoos for listed species, it still provides the most comprehensive current summary of these activities across AZA. Because of the specific focus of these surveys, the responses would also exclude education programs that do not directly target the local communities in the species’ native range. Therefore our analysis leaves out many of the conservation-oriented education projects carried out by zoos, which can also have significant impacts on achieving biodiversity conservation^21^.

We queried the database of field conservation and research survey responses for references to ESA-listed species in the project titles, descriptions, or the selected focal species. We tallied the number of conservation project submissions, representing unique combinations of institutions, projects, and species. That is, the same project may involve multiple institutions, and we count these as unique projects for each institution. This is because each institution may submit the project under a different name or description, thereby making it difficult to consistently delineate unique projects. Between 2013–2015, 142 AZA institutions reported a total of 644 active conservation projects involving 74 ESA-listed, U.S. terrestrial and avian species (23 mammals, 21 birds, 12 amphibians, 11 reptiles, and 7 invertebrates). Of these, 50 are currently listed as Endangered and 24 as Threatened. Although 54 of the 74 listings have finalized recovery plans, only 18 of those plans mentioned zoos as responsible parties for recovery actions.

Similar to the actions from recovery plans, we assigned each zoo project from the survey data to one or more of the 11 keywords representing different types of conservation activities (Table 1). Of the 786 keywords assigned, most were related to research (25.2%), monitoring/assessments (17.6%), population augmentation (16.0%), and managing assurance populations (12.7%; Fig. 1B). Fundraising directed to recovery programs or conservation organizations (for purposes unspecified in the survey response) accounted for 11.3% of the keywords. Projects related to education and outreach (targeting local communities in the species’ native range) accounted for 5.2% of the keywords, and all other keywords were used fewer than 3% of the time. Compared to the conservation actions described in recovery plans, zoos reported a smaller proportion of activities related to assurance populations, but a larger proportion related to monitoring and assessments, and to habitat creation/restoration/protection. This suggests that zoos are contributing more to *in situ* conservation projects than is recognized in recovery plans. Zoos also reported more fundraising projects than represented in recovery plans, and additionally reported several projects related to providing rescue, rehabilitation, or sanctuary facilities. Both data sources agreed that research made up a large proportion of the conservation activities in zoos, and that there was great variation in the types of research conducted. Research projects reported by zoos ranged from understanding the genetic structure of Hawaiian petrel (*Pterodroma sandwichensis*) populations, to measuring stress levels of Guam kingfishers (*Todiramphus cinnamominus*) in human care, to developing gene banking methods for black-footed ferrets (*Mustela nigripes*).

Comparing among taxonomic groups, the majority of zoo conservation projects involved listed mammal species (318 of 644 projects), and only 25 projects involved invertebrates. Although the distribution of projects among taxa is similar to a previous assessment of *in situ* conservation efforts by zoos around the world^16^, none of the mammalian species in our dataset were primates due to our focus on U.S. species. Based on the keyword categories we assigned to each project, we found *in situ* projects were most common for listed amphibians and invertebrates (Fig. 2B), and they primarily consisted of population augmentation projects. Knowledge and capacity projects were least common for amphibians and invertebrates, but they made up the largest proportion of projects for mammals, birds, and reptiles (consisting primarily of research projects). *Ex situ* projects made up less than 20% of all conservation projects reported by zoos for listed mammals, birds, and reptiles. Compared to the actions from recovery plans, a larger proportion of *in situ* projects were reported by zoos for all taxonomic groups, and a smaller proportion of *ex situ* projects were reported for all taxa except amphibians (Fig. 2).

**Figure 3:**
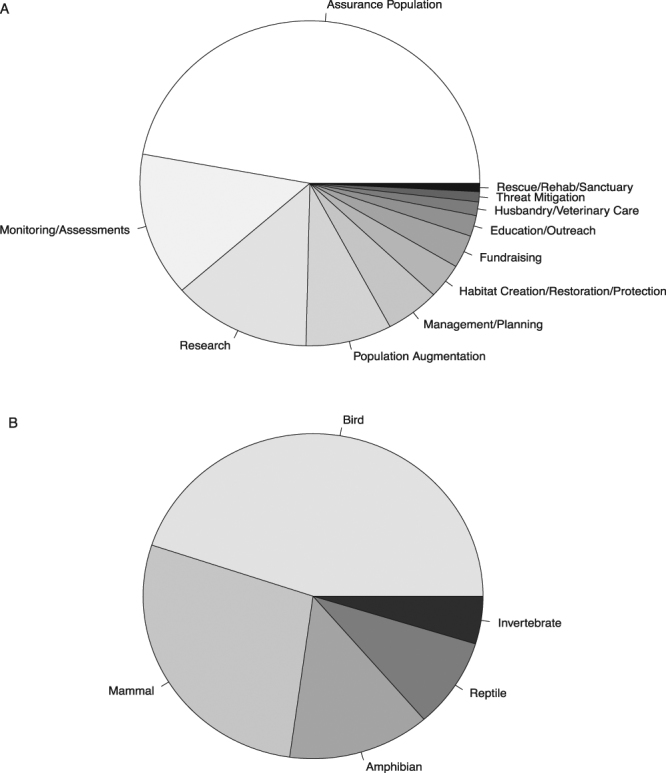
Spending by North American zoos and aquariums on conservation projects for species listed under the Endangered Species Act, as reported in the 2013–2015 Annual Report on Conservation and Science (ARCS) survey. The proportional spending (out of the total $28.9 M spent across 3 years) is shown by (**A**) project keyword and (**B**) taxonomic group.

We estimated the amount that AZA zoos spend on listed species by summing the project expenditures reported in the ARCS surveys. From 2013–2015, total spending on the reported field conservation and research projects specifically targeting the 74 ESA-listed species summed to $28.9 million, or on average $9.6 million per year. For context, the reported average spending per year on the same set of species in 2013–2015 was $146.4 million by all federal agencies, and $7.9 million by all state agencies^22–24^. Among the different types of conservation activities, the majority of funds were spent on assurance populations, followed by population monitoring and assessment and research (Fig. 3A). Comparing across taxa, expenditures were greatest on conservation projects for bird and mammal species (Fig. 3B).

### Listed Species with Managed Populations in Zoos and Aquariums

The recovery plans and AZA surveys provide an overview of the extent to which zoos currently contribute to recovering listed species. However, additional opportunities for conservation may exist, as a number of ESA-listed species have *ex situ* populations in zoos that are cooperatively managed. Since the 1980s, zoos have collaborated in managing the animals in their care through goal setting, cooperative breeding, and exchanging animals across institutions, with the aim of improving the health (e.g., demographic viability, genetic diversity) of those zoo animal populations^25,26^. In North America, cooperatively managed populations are those with a Species Survival Plan^®^ (SSP) program, which is implemented by AZA member institutions. SSPs may also coordinate the conservation, research, and educational initiatives among institutions to support *in situ* species recovery. These programs therefore represent opportunities for zoos to contribute further to conservation efforts, because they have an established management structure and working partnerships across institutions. Cooperative management also generates a great deal of species-specific knowledge on breeding, veterinary care, behavior, and demography, which can inform or facilitate conservation actions. For example, knowledge on how to breed animals successfully and to care for and rear offspring may be important for helping to improve reproduction of a threatened species. Further, the establishment of an SSP program demonstrates a long-term commitment to the species by multiple AZA institutions, which may be leveraged to promote engagement in and support for wild populations of the same species.

Overall, 143 of the 482 SSP programs (29.7%) were for ESA-listed species, representing 154 listings (which included separate listings for Distinct Population Segments or subspecies of the same species). The majority of these were for species listed as Endangered (83.4%) and as foreign (77.9%). Of the 387 listings for U.S. terrestrial and avian species, 36 (9.3%) currently have zoo populations managed by an SSP program. Interestingly, only 14 of the 54 species whose recovery plans specified roles for zoos had SSP populations, and 24 of the 74 species identified in the AZA surveys had SSP populations. Only 10 species overlapped across the three datasets, meaning they have recovery plans that specified a role for zoos, conservation projects reported by zoos in AZA surveys, and zoo populations managed by an SSP program. This finding suggests that an SSP program is not required for zoos to participate in recovery programs, and many zoos work with listed species outside of the SSP framework. On the other hand, there are additional SSP programs that could participate in that species’ recovery but currently do not.

**Figure 4:**
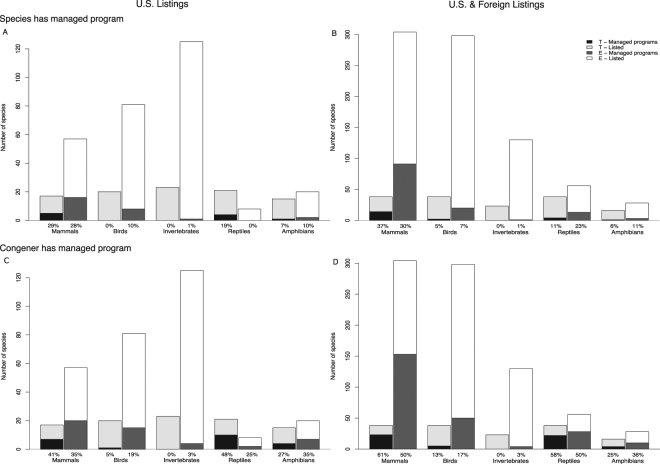
The proportion of terrestrial and avian animal species listed under the Endangered Species Act that have cooperatively managed populations in AZA-accredited zoos and aquariums, by taxonomic group and listing status (T = Threatened, E = Endangered). (**A**) The proportion of U.S. listings with managed programs for the listed species. (**B**) The proportion of U.S. and foreign listings with managed programs for the listed species. (**C**) The proportion of U.S. listings with managed programs for a congener of the listed species. (**D**) The proportion of U.S. and foreign listings with managed programs for a congener of the listed species.

Most of the SSP programs for listed species involved mammals, with existing programs for 21 of the 74 (28.4%) U.S. mammal listings (Fig. 4A). All other listed taxa were much less represented, especially invertebrates, for which the American burying beetle was the only listing (out of 148) with an SSP program. The picture was similar when including both U.S. and foreign listings, with 84 additional SSP programs for foreign-listed mammals, and a smaller number of additional SSP programs for foreign-listed birds and reptiles (14 and 13, respectively; Fig. 4B). In summary, the majority of SSP programs did not manage listed species, but those that did tended to focus on species that were more at risk (listed as Endangered rather than Threatened). There was also a taxonomic bias for SSP programs to focus on mammals and a geographic bias for non-U.S. species, many of which were native to African and Central American countries. Our results parallel findings from a previous study that zoo and aquarium collections favor larger vertebrate species^5^. However, the bias of SSP programs toward non-U.S. species contrasts with an earlier finding that zoos tended to focus on mammal and bird species that are native to economically developed countries^27^.

Zoos have the potential to contribute even further to species recovery, as shown by the number of listed species that have a congener with a managed SSP population in zoos (Fig. 4C,D). Management of a closely related species in the same genus produces valuable husbandry and biological information that may be useful for informing the conservation of the listed species. Institutions holding the congeners may also develop education programs or design exhibits to promote conservation actions for the closely related listed species. Additionally, since zoos already have the resources and facilities to house a closely related species, it may be possible for those institutions to house the more threatened species instead, if *ex situ* breeding or rehabilitation is deemed beneficial (of course, species-specific behaviors and requirements will determine the extent to which that would be feasible, while threats and recovery strategies will determine the appropriateness of an *ex situ* breeding program). Across all taxa, there were SSP programs for the congeners of 70 out of 387 (18.1%) U.S. listings, and 299 out of 969 (30.9%) U.S. and foreign listings of terrestrial and avian species. In particular, there were managed programs for the congeners of 36.5% and 41.4% of U.S. listings for mammals and reptiles, respectively (Fig. 4C), and 51.5% and 53.2% of total (U.S. and foreign) listings for mammals and reptiles, respectively (Fig. 4D). This represents a significant body of knowledge and resources that could greatly enhance species recovery efforts, but have yet to be broadly utilized.

## Discussion

Our evaluation showed that zoos contribute to a diverse array of *in situ* and *ex situ* conservation efforts, and serve as important partners in the recovery of threatened species in the U.S. Zoo conservation activities (Table 1) spanned many of the conservation actions previously described^7^. Beyond maintaining *ex situ* populations^5^ and increasing public understanding of biodiversity^21^, zoos carry out many more *in situ* projects than typically recognized (though see Olive and Jansen^12^), including a large number of monitoring projects. We also found that zoos conduct a range of field- and zoo-based conservation research projects, which were nearly as numerous as *ex situ* breeding efforts (Fig. 1). Biodiversity monitoring and research both help to support successful species recovery, but they are not commonly viewed as significant ways in which zoos contribute to conservation. Our findings support earlier studies that showed these critical conservation actions are increasingly being funded or conducted by NGOs^28,29^, including zoos.

However, additional opportunities exist. We found that similar to zoo holdings overall^27^, managed SSP populations currently focus on non-threatened species. Among listed species, however, managed programs do tend to prioritize species that are more at risk of extinction. There are many considerations that determine the selection of species for zoo exhibits, and management programs are increasingly including conservation status in their decision-making. However, if a species is especially difficult to house, cannot reproduce successfully, or has low survivorship in zoos, then establishing *ex situ* populations may not be feasible or worthwhile. Further, there are ways to contribute to conservation even if zoos are managing the less at-risk species that are closely related to a threatened species, as discussed above.

U.S. zoos may also increase their conservation efforts by managing more native threatened species, as our results showed a tendency for SSP programs to focus on foreign-listed species. *Ex situ* populations would ideally be established in the species’ native range^2^, but currently >90% of the U.S. listed avian and terrestrial species do not have an SSP population in North American zoos. Further research is needed to evaluate whether and the extent to which those listed species would benefit from *ex situ* population management. Zoos are also carrying out relatively few education and outreach programs that directly impact listed species in the wild (Fig. 1B). By including more native threatened species, zoos could develop associated education and outreach programs to engage the community most likely to impact the species and promote direct conservation actions. Of course, zoo education programs that do not directly affect wild populations are still valuable^21^, and we reiterate that our review did not summarize the magnitude of those existing efforts.

Finally, our findings suggest a need for greater coordination across zoos and better engagement with other conservation science partners. For example, 40 institutions reported working on various field conservation and research projects for the polar bear in the AZA surveys, but it is unclear the extent to which these efforts were coordinated to maximize their effectiveness. Only 5 recovery plans (for 5 species) named two or more zoos as the responsible party for any recovery action, suggesting such coordination among zoos is infrequent or poorly represented in plans. Only a quarter of the recovery plan actions conducted by zoos involved either academic or NGO partners, although integrating efforts into larger collaborations could lead to better outcomes^29^. However, coordination with other conservation partners may be increasing, as more partnerships between zoos and academic institutions are being formed (*e*.*g*., Smithsonian-Mason School of Conservation, the Phoenix Zoo - Arizona State University conservation partnership, the Living Earth Collaborative). Other zoo partnerships supporting species recovery include concentrated breeding centers and consortiums such as the Conservation Centers for Species Survival (C2S2), and AZA’s SAFE: Saving Animals From Extinction, a conservation framework launched in 2015 that prioritizes collaboration^14^. There are also efforts to integrate *ex situ* and *in situ* species management through the IUCN Conservation Planning Specialist Group’s One Plan Approach^30,31^.

In this assessment we focused on terrestrial and avian species listed under the ESA. Thus, the role of zoos in helping to conserve marine animals, plants, and species with other risk statuses remain to be examined. Additionally, further research is needed to evaluate the impacts of the many zoo conservation projects^18^, which could inform and improve future efforts. In summary, our study highlights the wide-ranging conservation actions conducted by North American zoos, and identify opportunities for better integration with the broader conservation community. By evaluating the current role of zoos in species conservation, our study provides a better understanding of the expertise, resources, and opportunities that zoos can offer as one of the many necessary partners in recovering threatened species.

### Data availability

The recovery plan data analyzed in the current study are included in the Supplementary Information (Table S2). The AZA survey data, except financial information, are available on AZA’s website (http://www.aza.org/field-conservation; http://www.aza.org/research-and-science). Additional data are available from the corresponding author on reasonable request.

## Electronic supplementary material

Supplementary information

Table S2

## References

[1] McNeely, J. A. *Expanding Partnerships in Conservation* (Island Press, 1995).

[2] Conde DA, Flesness N, Colchero F, Jones OR, Scheuerlein A (2011). An emerging role of zoos to conserve biodiversity. Science.

[3] Zimmermann, A., Hatchwell, M., Dickie, L. A. & West, C. *Zoos in the 21st Century: Catalysts for Conservation?* (Cambridge University Press, 2007).

[4] Minteer, B. A., Maienschein, J., & Collins, J. P. *The Ark and Beyond* (The University of Chicago Press, 2018).

[5] Balmford A, Mace GM, Leader‐Williams N (1996). Designing the ark: Setting priorities for captive breeding. Conservation Biology.

[6] Salafsky N (2008). A standard lexicon for biodiversity conservation: Unified classifications of threats and actions. Conservation Biology.

[7] Conservation Measures Partnership. Classification of conservation actions and threats. Version 2.0, http://cmp-openstandards.org/tools/threats-and-actions-taxonomies/ (2016).

[8] Snyder NFR (1996). Limitations of captive breeding in endangered species recovery. Conservation Biology.

[9] Scott JM (2005). Recovery of imperiled species under the Endangered Species Act: the need for a new approach. Frontiers in Ecology and the Environment.

[10] Conway WG (2011). Buying time for wild animals with zoos. Zoo Biol..

[11] Lacy RC (2013). Achieving true sustainability of zoo populations. Zoo Biology.

[12] Olive A, Jansen K (2017). The contribution of zoos and aquaria to Aichi Biodiversity Target 12: A case study of Canadian zoos. Global Ecology and Conservation.

[13] Redford KH, Jensen DB, Breheny JJ (2012). Integrating the captive and the wild. Science.

[14] Grow, S., Luke, D. & Ogden, J. Saving Animals from Extinction (SAFE): Unifying the conservation approach of AZA-accredited zoos and aquariums. [Minteer, B., Maienschein, J. & Collins, J. (eds)] The Ark and Beyond 9, 122–128 (The University of Chicago Press, 2018).

[15] Zimmermann, A. The role of zoos in contributing to *in situ* conservation. [Kleiman, D. G., Thompson, K. V. & Baer, C. K. (eds)] Wild Mammals in Captivity: Principles and Techniques for Zoo Management. 23, 281–287 (The University of Chicago Press, 2010).

[16] Gusset M, Dick G (2010). ‘Building a Future for Wildlife’? Evaluating the contribution of the world zoo and aquarium community to *in situ* conservation. International Zoo Yearbook.

[17] Evans, D. M. *et al*. Species recovery in the United States: Increasing the effectiveness of the Endangered Species Act. *Issues in Ecology* **20** (2016).

[18] Mace, G. M. *et al*. Measuring conservation success: assessing zoos’ contribution. [Zimmermann, A., Hatchwell, M., Dickie, L. A. & West, C. (eds)] Zoos in the 21st Century: Catalysts for Conservation. 21, 322–342 (Cambridge University Press, 2007).

[19] Ballantyne R, Packer J, Hughes K, Dierking L (2007). Conservation learning in wildlife tourism settings: lessons from research in zoos and aquariums. Environmental Education Research.

[20] USFWS. Revised Hawaiian Forest Birds Recovery Plan, https://ecos.fws.gov/docs/recovery_plan/060922a.pdf (2006).

[21] Moss A, Jensen E, Gusset M (2017). Impact of a global biodiversity education campaign on zoo and aquarium visitors. Frontiers in Ecology and the Environment.

[22] USFWS. Federal and State Endangered and Threatened Species Expenditures – Fiscal year 2013, https://www.fws.gov/ENDANGERED/esa-library/pdf/2013.EXP.FINAL.pdf (2015).

[23] USFWS. Federal and State Endangered and Threatened Species Expenditures – Fiscal year 2014, https://www.fws.gov/ENDANGERED/esa-library/pdf/20160302_final_FY14_ExpRpt.pdf (2016).

[24] USFWS. Federal and State Endangered and Threatened Species Expenditures – Fiscal year 2015, https://www.fws.gov/ENDANGERED/esa-library/pdf/2015_Expenditures_Report.pdf (2017).

[25] Ballou, J. D. *et al*. Demographic and genetic management of captive populations. [Kleiman, D. G., Thompson, K. V. & Baer, C. K. (eds)] Wild Mammals in Captivity: Principles and Techniques for Zoo Management. 19, 219–252 (University of Chicago Press, 2010).

[26] Henson, P. American zoos: a shifting balance between recreation and conservation. [Minteer, B., Maienschein, J. & Collins, J. (eds)] The Ark and Beyond 5, 65–76 (The University of Chicago Press, 2018).

[27] Martin TE, Lurbiecki H, Joy JB, Mooers AO (2014). Mammal and bird species held in zoos are less endemic and less threatened than their close relatives not held in zoos. Anim Conserv.

[28] Bakker VJ (2010). The changing landscape of conservation science funding in the United States. Conservation Letters.

[29] Lindenmayer DB (2012). Improving biodiversity monitoring. Austral Ecology.

[30] Byers O, Lees CM, Wilcken J, Schwitzer C (2013). The One Plan Approach: The philosophy and implementation of CBSG’s approach to integrated species conservation planning. WAZA Magazine.

[31] Traylor-Holzer, K., Leus, K. & Byers, O. Integrating *ex situ* management options as part of a One Plan Approach to species conservation. [Minteer, B., Maienschein, J. & Collins, J. (eds)] The Ark and Beyond 10, 129–141 (The University of Chicago Press, 2018).

