# Supplementary material for: Evaluating the Contribution of North American Zoos and Aquariums to Endangered Species Recovery
Source: Sci Rep. 2018 Jun 28;8:9789. doi: 10.1038/s41598-018-27806-2 (PMC6023936; doi:10.1038/s41598-018-27806-2)
Supplement: Supplementary file 1 — Supplementary information [file 41598_2018_27806_MOESM1_ESM.pdf]

Evaluating the Contribution of North American Zoos and Aquariums to Endangered Species Recovery

Judy P. Che-Castaldo, Shelly A. Grow, and Lisa J. Faust

Supplementary Information

Table S1. Complete list of zoos and aquariums that were listed as the responsible party for at least one recovery action in recovery plans for species listed under the U.S. Endangered Species Act as of September 2016. Italicized names do not identify a specific institution to our knowledge. Each institution is listed as it was identified in the recovery plan, and thus may not reflect its official or current institutional name.

**Zoo Instituation**
Association of Zoos & Aquariums (formerly American Association of Zoological Parks and Aquariums), or "Species Survival Plan® (SSP) program partners"
Arizona-Sonora Desert Museum
Boise Zoo
Burnet Park Zoo (Rosamond Gifford Zoo)
Cabrillo Aquarium
Chicago Zoological Society
Cincinnati Zoo and Botanical Garden
Columbus Zoo
Dallas Aquarium
Detroit Zoo
Florida Aquaria
Fort Worth Zoo
Gladys Porter Zoo
Honolulu Zoo
Living Seas
Long Beach Aquarium of the Pacific
Los Angeles Zoo
Lowry Park Zoo
Memphis Zoo
Miami Seaquarium
Monterey Bay Aquarium
Mote Marine Laboratory
New England Aquarium
Oregon Zoo
Phoenix Zoo
Point Defiance Zoo
Roger Williams Park Zoo
San Diego Wild Animal Park
San Diego Zoo Safari Park

San Diego Zoological Society
Santa Barbara Zoo
Sea World Florida & California
Seattle Aquarium
South Florida Museum
Tennessee Aquarium Research Institute
Toledo Zoo
USFWS Aquaria
Vancouver Aquarium
Wildlife Conservation Park/Bronx Zoo
Zoological Park

Table S2. Recovery plan data accessed from the USFWS Recovery Plan Ad Hoc Report database (http://ecos.fws.gov/ecp0/ore-input/ad-hoc-recovery-actions-public-report-input) in September 2016, consisting of information for all plans that contain recovery actions listing a zoo, aquarium, or Association of Zoos & Aquariums (AZA) as the responsible party.

(in separate .csv file)
